# Supplementary material for: Climate variability and Germanic settlement dynamics in the Middle Danube region during the Roman Period (1st–4th Century CE)
Source: PLoS One. 2025 Jul 16;20(7):e0325139. doi: 10.1371/journal.pone.0325139 (PMC12266394; doi:10.1371/journal.pone.0325139)
Supplement: Attachment — Submitted filename: Response to Reviewers.docx [file pone.0325139.s002.docx]

Mgr. Marek Vlach, Ph.D.

Head of the Research Center for the Roman Period and Migration Period

Institute of Archaeology of the Czech Academy of Sciences, Brno
Čechyňská 363/19, 602 00 Brno, Czech Republic

[vlach@arub.cz](mailto:vlach@arub.cz)

+420 608 977 267

December 28^th^, 2024

Dear Dr. Biehl,

We would like to express our sincere gratitude to the reviewers for their insightful comments regarding the manuscript, **Climate Variability and Germanic Settlement Dynamics in the Middle Danube Region during the Roman Period (1st–4th Century CE)**. We have revised the manuscript to address the raised concerns, objections, and suggestions of individual reviewers below. Also, the manuscript formatting has been adjusted to comply with the journal standards.

**Reviewer 1**

*This interdisciplinary study is highly stimulating and most suitable for publication in PLOS1! The background and motivation are clearly outlined in the "Introduction", the data used, and methods applied are described carefully, and the results are critically discussed. The study has the potential provoke further investigations at the climate-human interface, and encourage scholars from the natural and social sciences and the humanities to join forces. Although I would have written parts of the manuscript slightly differently, I recommend acceptance as it is.*

**Response**: Reviewer 1 provides a positive reflection, but as a member of the author team of the paper, there is a conflict of interest, and no further reactions will be made in this regard.

**Reviewer 2**

*The study under review entitled “Climate variability and Germanic settlement dynamics in the Middle Danube region during the Roman Period (1st–4th Century CE)” explores the relationship between climate and human societies by examining the Germanic populations in the Middle Danube region during the Roman period. While the authors utilize an interdisciplinary approach combining palaeoclimatic, archaeological, and documentary datasets, the work suffers from significant methodological and interpretive shortcomings. Below, I outline the major issues with the study.*

*The integration of palaeoclimatic and archaeological data is commendable, offering a valuable attempt at interdisciplinary analysis. The spatiotemporal overlap of datasets allows for a nuanced exploration of the relationship between environmental and societal factors.*

*However, the authors take for granted that climate significantly influenced settlement structures without critically examining the validity of this hypothesis. The study lacks an open-ended inquiry and instead seeks to quantify an assumed climate-driven relationship.*

*The authors narrowly attribute settlement changes to climatic variability while ignoring alternative explanations. This one-dimensional approach undermines the study’s credibility, as non-climatic factors such as warfare, economic crises, and demographic pressures likely played substantial roles. Moreover, the study conflates correlation with causation. For instance, the claim that 77% of settlements were “active” during favourable agroclimatic conditions assumes a direct causal link, ignoring other potential drivers of settlement activity.*

**Response**: We appreciate the reflections on the manuscript. However, we feel that up to a certain proportion, the statement by the reviewer about the uncritical acceptance and unilateral endorsement of climate-driven changes of the Germanic societies of the Middle Danube region, while ignoring the other potential factors, does not entirely reflect the reality of the manuscript. On multiple occasions (lines 45-58, 320-326, 482-484), the role and potential of multiple other drivers of changes (e.g., documented military conflicts, social or economic development changes) are considered to provide unbiased insight. Particularly, the significant changes during the 2nd half of the 2nd century CE (time block 150-200 CE) cannot be associated with the agroclimatic, and the extensive conflict of the Marcomannic wars could be large-scale decreases in settlement structure size (lines 278-281, 427-442).

The study was primarily conceived to provide a deeper insight into the human-climate responses recently made available by the intersection of the two specific datasets. As a result, quantifying the potential impact of climate change on the anthropogenic context may provide substantiation for assumptions about other drivers of change. Simultaneously, the recorded co-occurrences of the documented changes in settlement structures and in agroclimatic reconstructions outside this particular warfare period are not absolute and they reach the percentages that allow for other drivers of change and forcings (lines 449-454). Nevertheless, the clues for their identification are not always apparent or present in archaeological and historical data. The objected passage in the text regarding the 77% of settlements in an ‘active’ stance during the more favourable climate conditions is provided as a general trend which has oscillating propagation in individual time periods in consideration, and it still leaves over 20% of other causes and drivers of change. The variability in incidence of climate-driven changes in several time blocks (foremost 0-50 and 250-300 CE). However, the text has been modified to provide more multilateral reflection on the potential drivers of change, whether internal or external. However, the study's findings indicate a non-accidental reliance of the Germanic subsistence on agroclimatic conditions.

*The historical and geopolitical background of the Marcomanni in the second century CE is insufficiently addressed. Key aspects such as the societal, economic, and demographic dynamics of the Marcomannic settlement zone are mentioned but not explored in detail. The study fails to consider the impact of critical events like the Antonine Plague, shifting trade patterns, and economic disruptions in the Roman Empire, all of which likely influenced settlement dynamics.*

**Response**: As suggested by the reviewer, further information on the historical context of the key period was provided (lines 397-408). The role of the Antonine Plague as one of the drivers of the upcoming ‘3^rd^-century crisis’ had, according to the present state of knowledge, the most significant impact within the extent of the Roman empire with high population densities, which further influenced various segments of the Roman context (economy). However, the considerably lower population densities within the Germanic study region, with the absence of agglomerations, allow only limited impact on the demography context. The spread of contagion could have been mediated by the Roman armies operating on the Germanic territories during the offensive phases of the Marcomannic wars, which are considered effective vectors of disease spread in the Roman empire.

*The study uncritically adopts the concept of the “Roman Climate Optimum” and its dating from Kyle Harper’s The Fate of Rome (2017). This work, while popular, has been heavily criticized for oversimplifying complex historical and scientific issues. The term "Late Roman Period" is left undefined, leaving readers uncertain about its chronological framework.*

**Response**: The concept of the ‘Roman Climate Optimum’ has been reflected in the study as a global underlying phenomenon, which is generally consistent with other studies, and *Harper 2017* is not an exception. Yet, further references and reflections on the phenomenon were provided in the text (lines 377-385). Simultaneously, there are no further considerations on this phenomenon in the manuscript that would require further elaboration and examination.

Conversely, there is certainly a point in the necessity of providing temporal specification to the term ‘Late Roman Period’, which is embedded foremost in the chronological frameworks of the Roman Period archaeology of Germanic parts of Europe, and this deficiency was accordingly rectified in the text (lines 85-86).

*Moreover, the study fails to situate its findings within the broader Mediterranean context. The observed settlement patterns—growth during the 1st and early 2nd centuries CE, decline in the late 2nd and early 3rd centuries, and stabilization thereafter—are not unique to the Germanic settlement zone but reflect broader trends across the Roman world. A more thorough historical contextualization could have provided critical insights into shared patterns of societal response to changing conditions.*

*The study’s exclusive focus on climatic drivers, lack of historical contextualization, and methodological flaws render its conclusions unconvincing. While the interdisciplinary framework holds promise, the analysis is undermined by a narrow, uncritical approach and insufficient consideration of alternative explanations. For these reasons, I recommend rejection of this study in its current form.*

**Response**: It is particularly the explicit focus on the spatial domain of the intersection of the two input datasets, on which exploration is the focal point of the study set. Therefore, the comparison with the clearly heterogeneous context of the Mediterranean is not particularly relevant for the study's objectives (lines 224-226).

We are convinced that the emerging objections have been reflected, addressed and argued properly. The respective parts of the manuscript have been extended to reflect the features in the reconstructed temporal patterns and highlight their differences. As well, respective changes have been implemented into the manuscript to balance the ‘open-ended’ inquiry factor, which is, in such studies, certainly a natural component due to embedded uncertainties in archaeological and documentary sources of information.

**Reviewer 3**

*Very interesting paper - well conceived and executed, was fun to read. Some things to note for improvement:*

*1. Some English language/grammar corrections needed in the abstract, the rest is fine.*

**Response**: The language issues in the abstract have been corrected.

*2. With regard to the archaeological data that has been synthesized (pg 5), it should be mentioned briefly by which methods the original data were obtained (e.g., intensive survey vs extensive survey, research excavation vs rescue excavation, etc.) as this can have a major effect the detection of sites and their distribution and therefore the reliability of the data.*

**Response**: This is clearly a valid point, and the text was further elaborated in the section concerning the nature and quality of input archaeological information (lines 119-129).

*3. Although biases and uncertainties in the archaeological and climatological data are noted, there is no acknowledgement of the possible biases in the historical documentary sources. This is worth mentioning in the text.*

**Response**: As in the previous point, this is also a clearly valid suggestion, and the respective section has been supplemented (lines 137-148).

On behalf of the authors

Marek Vlach
