# Supplementary material for: Climate variability and Germanic settlement dynamics in the Middle Danube region during the Roman Period (1st–4th Century CE)
Source: PLoS One. 2025 Jul 16;20(7):e0325139. doi: 10.1371/journal.pone.0325139 (PMC12266394; doi:10.1371/journal.pone.0325139)
Supplement: Attachment — Submitted filename: Response_to_Reviewers_auresp_2.docx [file pone.0325139.s003.docx]

Mgr. Marek Vlach, Ph.D.

Head of the Research Center for Protohistoric Archaeology

Institute of Archaeology of the Czech Academy of Sciences, Brno
Čechyňská 363/19, 602 00 Brno, Czech Republic

[vlach@arub.cz](mailto:vlach@arub.cz)

+420 608 977 267

April 10^th^, 2025

Dear Dr. Biehl,

We very much appreciate the reviewers' effort and time given to the manuscript **Climate Variability and Germanic Settlement Dynamics in the Middle Danube Region during the Roman Period (1st–4th Century CE)**. We have revised the manuscript and address the remaining concerns and objections. As required, there have been made corrections in the reference list and the newly added references are listed below.

Vlach M. Germanic communities of the ‘Marcomannic’ settlement zone: structure and dynamics. Brno: Institute of Archaeology of the Czech Academy of Sciences, Brno; 2024.

Domański G. Die Bevölkerungszunahme in Mitteleuropa und die Gründe für den Ausbruch der Markomannenkriege. In: Tejral J, Stuppner A, Friesinger H, editors. Markomannenkriege Ursachen und Wirkungen 6 Internationales Symposium "Grundprobleme der frühgeschichtlichen Entwicklung im nördlichen Mitteldonaugebiet", Wien, 23-26 November 1993. Spisy Archeologického ústavu AVČR, Brno. Brno: Archeologický ústav AV ČR, Brno; 1994. p. 109-14.

Groenewoudt BJ, Van Lanen RNJ. Diverging decline. Reconstructing and validating (post-)roman population trends (AD 0-1000) in the rhine-meuse delta (The Netherlands). European Journal of Post-Classical Archaeologies. 2018;8:189-218.

Maddison A. Contours of the world economy, 1-2030 AD: essays in macro-economic history. 1st ed. Oxford: Oxford University Press; 2007.

Frier BW. Demography. In: Bowman AK, Garnsey P, Rathbone D, editors. The Cambridge Ancient History: Volume 11: The High Empire, AD 70–192. The Cambridge Ancient History. 11. 2 ed. Cambridge: Cambridge University Press; 2000. p. 787-816.

Haldon JF, Roberts N, McCormick M, Doonan OP, Elton H, Ladstätter S, et al. The Climate and Environment of Byzantine Anatolia: Integrating Science, History, and Archaeology. Journal of Interdisciplinary History. 2014;45(2):113-61. doi: 10.1162/JINH_a_00682.

Schmidt I, Hilpert J, Kretschmer I, Peters R, Broich M, Schiesberg S, et al. Approaching prehistoric demography: proxies, scales and scope of the Cologne Protocol in European contexts. Philos Trans R Soc Lond B Biol Sci. 2021;376(1816):20190714. Epub 20201130. doi: 10.1098/rstb.2019.0714. PubMed PMID: 33250025; PubMed Central PMCID: PMCPMC7741091.

Scheidel W, editor. Debating Roman demography. Leiden-Boston: Brill; 2001.

Duncan-Jones R. Structure and scale in the Roman economy. Cambridge-New York: Cambridge University Press; 1990. xvi, 245 p. p.

Scheidel W, editor. The science of Roman history : biology, climate, and the future of the past. Princeton: Princeton University Press; 2018.

Bagnall RS, Frier BW. The Demography of Roman Egypt. Cambridge: Cambridge University Press; 1994.

Scheidel W. Death on the Nile : disease and the demography of Roman Egypt: Brill; 2001. 1 online resource p.

Elliott CP. The Antonine Plague, Climate Change and Local Violence in Roman Egypt Past & Present. 2016;231(1):3-31. doi: 10.1093/pastj/gtv058.

Palmisano A, Bevan A, Shennan S. Comparing archaeological proxies for long-term population patterns: An example from central Italy. Journal of Archaeological Science. 2017;87:59-72. doi: 10.1016/j.jas.2017.10.001.

Verhagen P, Joyce J, Groenhuijzen MR, editors. Finding the limits of the limes: modelling demography, economy and transport on the edge of the Roman empire. Cham: Springer; 2019.

Bevan A. Big Questions for Large, Complex Datasets: Approaching Time and Space Using Composite Object Assemblages. Internet Archaeology. 2012;31.

Jongman WM. The Economic Archaeology of Roman Economic Performance. In: Verhagen P, Joyce J, Groenhuijzen MR, editors. Finding the Limits of the Limes: Modelling Demography, Economy and Transport on the Edge of the Roman Empire. Cham: Springer; 2019. p. 95-107.

Contreras D, Guiot J, Suarez R, Kirman A. Reaching the human scale: A spatial and temporal downscaling approach to the archaeological implications of paleoclimate data. Journal of Archaeological Science. 2018;93:54-67. doi: 10.1016/j.jas.2018.02.013.

Lenz KH, Berke H, Heußner Br. Siedlungen der römischen Kaiserzeit auf der Aldenhover Platte. Köln-Bonn: Habelt; 1999.

Karasaridis A, Chalupa A. Comparative SIR/SEIR modeling of the Antonine Plague in Rome. PLoS One. 2025;20(2):e0313684. Epub 20250213. doi: 10.1371/journal.pone.0313684. PubMed PMID: 39946392; PubMed Central PMCID: PMCPMC11824979.

Becker M, Bauer S, Meller H. Das Fürstengrab von Gommern. Halle: Landesamt für Denkmalpflege und Archäologie Sachsen-Anhalt, Landesmuseum für Vorgeschichte; 2010.

Droberjar E. Neue Erkenntnisse zu den Fürstengräbern der Gruppe Haßleben-Leuna-Gommern in Böhmen. Přehled výzkumů. 2007;48:93-103.

Klčo Mn, Krupa Vr. German graves from Roman times from Krakovany - Stráže. Piešt'any, Slovakia: Balneological Museum; 2003.

**Reviewer 2**

*The authors have made an effort to address several of my critiques, but not all have been fully resolved. They have worked to clarify the role of non-climatic factors in settlement changes, adding references to historical events and refining their interpretation of climate’s influence. However, their response still leans heavily on climate as a primary driver, without fully engaging in a more open-ended analysis of the broader social, economic, and geopolitical forces at play. While they acknowledge alternative explanations, these remain secondary rather than being genuinely integrated into the discussion.*

We agree that climate only represents one part in the complex topic that is demographic changes. We also believe that much of the debate on climatic influences on Germanic and Roman population dynamics have been much too simplified due to generalisation and a lack of local (or even) regional records. However, our comparison is cemented for relatively well-defined spatial conditions. Going back 1500-2000 years, the available climate proxy records are extremely limited – especially if limiting oneself to annually-resolved estimates. As a consequence, long tree-ring chronologies from the European Alps and ice-core records from Greenland have been assumed to directly represent conditions in regions from the Mediterranean to the British Isles. The reality on the ground is likely much more complex. However, our study only compares proxy data that have been robustly verified for the past 900+ years to archaeological information from the area from which the reconstructions stem. From multiple perspectives, we believe this to be a unique situation and comparison, which aligns with previous calls (e.g., Jongman 2019) for more detailed empirical evidence and scientific archaeology. These reflections have been also incorporated to the text (e.g., lines 319-325, 446-451, 533-536, 592-599).

*Their expansion on the historical and geopolitical background of the Marcomannic period, including the Antonine Plague, is a welcome addition. However, their argument that the plague had minimal impact on Germanic populations due to lower population density seems overly simplistic. While they recognise the potential role of Roman armies in spreading disease, they stop short of fully exploring how pandemics can affect frontier societies beyond direct population loss.*

We acknowledge this objection as fully justified and there has been provided further elaboration on the subject reflecting the potential of the epidemic impact to the studied context (lines 456-468).

*Regarding the use of the "Roman Climate Optimum," adding references helps provide context, but the authors do not critically engage with the debate surrounding this concept. Simply citing additional studies does not address the concern that the framework itself is contested and may oversimplify historical complexities.*

The revised version has attempted to present a more nuanced description of the complexities the reviewer mentioned. We do not disagree with these thoughts, and although our study focuses on the agroclimatic component of variability, we do not consider it climatic determinism. Also, the concept of the ‘optimum’ does not play any substantial interpretative role in the study as the variability is more acknowledged for the regionally resolved development patterns (lines 390-397).

*Their rejection of a broader Mediterranean comparison is also unconvincing. While I understand their regional focus, the settlement patterns they describe are not unique to the Middle Danube. Acknowledging parallels elsewhere would have strengthened their argument rather than detracted from it.*

At this point we do not believe that the comparison is practically meaningful – in terms of cumulative uncertainty. However, this assertion should not be seen as a dismissal of the reviewer’s comment. We fully agree that Mediterranean events affected Roman strategies regarding the frontiers, but when considering that the relationship is likely lagged in time, it easily becomes an exercise in “cherry-picking” without clear quantification. Parallels with other regions (including other *limes* areas) suffer from the lack of high-resolution and high-quality palaeoclimatic estimates. Because of the multiple sources of uncertainty that exists in comparisons (e.g., some of the proxy records from the Mediterranean region that have previously been used lack the temporal resolution to provide meaningful basis for caparison), we argue that our approach, while acknowledging these limitations, offers one perspective of a crucial period of European history. Of course, this is not meant to be the final word on the topic. Simultaneously, further parallels to development patterns in other parts of the Germanic and Roman worlds have been supplemented (lines 222-240).

*Overall, while the revisions improve the manuscript in some areas, key methodological and interpretive concerns remain. The study still leans too heavily on climate determinism, and alternative explanations, though now mentioned, are not fully explored. The changes represent progress, but they do not completely resolve the fundamental issues I raised.*

We hope that the responses here and the additional changes to the manuscript will represent sufficient reflections and a compromise acceptable to all parties.

References:

Jongman, W.M. (2019) The economic archaeology of Roman economic performance. In Verhagen, P., Joyce, J., Groenhuijzen, M.R. (Eds.) Finding the Limits of the Limes. Springer, Amsterdam.

On behalf of the authors

Marek Vlach
